# The impact of a specialist home-visiting intervention on the language outcomes of young mothers and their children: a pragmatic randomised controlled trial

**DOI:** 10.1186/s40359-022-00926-1

**Published:** 2022-09-23

**Authors:** Cerith S. Waters, Rebecca Cannings-John, Susan Channon, Fiona Lugg-Widger, Mike Robling, Amy L. Paine

**Affiliations:** 1 Cardiff University Centre for Human Developmental Science, School of Psychology, Cardiff University, Park Place, Cardiff, CF10 3AT Wales UK; 2 Centre for Trials Research, Cardiff University, Neuadd Meirionnydd, Heath Park, Cardiff, CF14 4YS Wales UK

**Keywords:** Young motherhood, Family Nurse Partnership, Mother–child interaction, Language, Randomized-controlled trial

## Abstract

**Background:**

Young mothers are more likely to provide a suboptimal early language environment for their children who in turn show impairments in their language development, yet few studies have used observational methods to assess the effectiveness of home-visiting programmes in improving the language outcomes of young mothers and their children. The Family Nurse Partnership (FNP) is a licensed home-visiting intervention developed in the USA and introduced into practice in England. The intervention involves up to 64 structured home visits from early pregnancy until the child's second birthday by specially recruited and trained Family Nurses. We assessed the effectiveness of FNP in improving the language outcomes of first-time teenage mothers and their infants.

**Method:**

We conducted a pragmatic, non-blinded, randomised controlled trial to test whether the FNP programme improved mothers’ and children’s language production at 24 months postpartum. Eligible participants were nulliparous, aged 19 years or younger, and were recruited at less than 25 weeks’ gestation from community midwifery settings (Country). Pregnant young mothers were randomly assigned to FNP plus usual care (n = 243) or usual care alone (n = 233). At 24 months postpartum, mother–child dyads were observed during a standardised free-play task with their first-born child and features of their language production was coded. Data was analysed using multi-level modelling; linear or poisson/negative binomial regression models were used as appropriate.

**Results:**

A small effect of FNP on mothers’ productive language was detected, where mothers in the FNP group demonstrated higher mean length of utterances than mothers who received usual care alone, mean difference (adjusted by minimisation variables and by site, linear regression) = 0.10, p < .05, 95% CI (0.004–0.20), d = .18. No differences were detected between groups regarding other characteristics of maternal language or children’s language outcomes.

**Conclusion:**

This observational study conducted within the context of a randomised-controlled trial suggests that the FNP home-visiting programme may have a small, but potentially important impact on young mothers’ speech to their toddlers. Exploratory analyses identified family environment, maternal, and child related predictors of the language outcomes of young mothers and their offspring.

*Trial registration* This trial is registered with ISRCTN, number ISRCTN23019866, 20/04/2009.

**Supplementary Information:**

The online version contains supplementary material available at 10.1186/s40359-022-00926-1.

## Background

Teenage parenthood remains high on the political and public health agenda and is associated with a range of adverse outcomes for both mother and child. Becoming a parent during the teenage years predicts long-term poverty, family instability and mental ill-health [1]. Children born to teenage mothers display suboptimal language development, score lower on standardized tests of cognitive ability, and experience elevated rates of behavioural and emotional problems [1–3]. These childhood difficulties persist into later life leading to higher rates of educational under-achievement, criminal convictions, and mental health problems [4, 5]. Consequently, early intervention programmes have been developed to improve the lives of teenage mothers and their offspring [6].

Intervention programmes that begin early in a child’s life and place emphasis on enhancing parenting skills and promoting children’s language development can be effective in improving children’s longer-term outcomes [6, 7]. Language acquisition is a key developmental milestone that is crucial for a child’s subsequent cognitive, behavioural, and socio-emotional development and is therefore a key target for intervention. Early language development is inextricably linked to the quality of the language environment provided by primary caregivers [8, 9], and is a key predictor of later reading proficiency, academic success, and psychosocial functioning [10]. Adolescent mothers’ own low verbal ability and sub-optimal parenting skills predict delays in their children’s language development [2, 3]. In some studies, interventions aimed at enhancing parent–child interactions and the quality and quantity of parental speech have been shown to be effective in improving children’s language and cognitive outcomes among adolescent mother–child dyads [7].

Home-visiting programmes that begin during pregnancy or shortly after childbirth and continue through the first one to two years of a child’s life represent one approach to improving parent–child interaction and the language skills of teenage mothers and their offspring. Home-visiting programmes are family-focused interventions that aim to improve multiple health, social, and educational outcomes for both mother and child. Evidence supporting the efficacy of home-visiting programmes in improving children’s language outcomes and mothers’ parenting skills is promising but inconsistent. In general, home-visiting programmes that begin in pregnancy and involve more frequent home visits report better parenting and child language outcomes [6, 11, 12]. In contrast, home-visiting programmes that begin after childbirth and involve less frequent home visits often fail to replicate the positive impact on children’s language development and mothers’ parenting skills [13]. There are, however, limitations to these broad conclusions, as not all home-visiting programmes that begin in pregnancy have demonstrated a positive impact on children’s language abilities [14]. Furthermore, assessments of mothers’ parenting skills tend to focus on maternal and/or home visitor ratings rather than independent evaluations of mother–child interaction [15]. An investigation of young mothers’ behaviour during interactions with their children may reveal processes that could be integrated into intervention programmes to improve children’s language and cognitive outcomes [16].

Originating in the USA, the *Nurse Family Partnership* (NFP) is one of the most well established home-visiting programmes. The seminal work of Olds and colleagues who developed the NFP reported mixed findings for children’s language and parenting outcomes. In a trial of NFP conducted in Elmira USA, NFP mothers, particularly unmarried teenagers, scored higher on the language stimulation scale of the HOME inventory than those who did not receive NFP [17]. In the Memphis trial, no effect of the programme was detected on mothers’ teaching behaviour at 24 months, however children born to nurse-visited mothers with low psychological resources were more communicative and responsive than their control-group counterparts [18]. By age six, nurse-visited children in the Memphis trial had better receptive vocabulary [12]. In the Denver trial, NFP delivered by paraprofessionals did not improve children’s directly assessed language outcomes or the quality of parent–child interaction [6, 19, 20]. In contrast, when the NFP was delivered by qualified nurses, significant improvements in children’s directly assessed language outcomes were observed, but only for the offspring of young mothers defined as low in psychological resources. Specifically, for children of mothers with low psychological resources, NFP significantly improved children’s receptive language skills at ages two and four years but not at six years [6, 12, 19, 20]. Significant improvements for nurse-visited families at age two years in the quality of parent–child interaction (mother-infant responsiveness) was observed for both high and low resource mothers [6]. At the age four follow-up the home environments of low resource mothers were more supportive of children’s learning [12, 19].

Given that the findings of Olds and colleagues were partially replicated across samples, and that the effectiveness of complex interventions does not always translate across contexts [21], it is important to examine the efficacy of NFP in improving the language environment provided by parents and children’s language outcomes in countries outside of the USA. The German adaptation of the original NFP programme, *‘Pro Kind’* failed to replicate the positive impact of NFP on children’s language outcomes and parent–child interaction [22]. Canadian and Dutch trials of NFP did not assess programme impact on children’s language ability or parent–child interaction [23, 24]. We conducted the evaluation of the NFP in England (the Building Blocks trial), which was commissioned by the United Kingdom Government (UK) and was licenced in the UK [25], as the *Family Nurse Partnership* (FNP). Analyses of the secondary outcomes revealed that by the time children were 24-months-old, mothers in the FNP trial arm reported their children’s language to be better developed when compared to families who received usual care alone. As a potentially important finding, it is now essential to provide in-depth and independent measures of children’s language development and maternal interactive behaviour [26].

Although young mothers’ language input is a primary source of variation in children’s early language ability [2, 3], little is known about other environmental factors that shape individual differences in the language skills of young mothers and their children. Therefore, the second aim of the current study was to examine individual differences in mothers’ and children’s language production. Young motherhood is associated with numerous risk factors that may contribute to reduced language input from the mother and poorer language development in her offspring [3, 7]. It is well-recognised that the social disadvantage associated with young motherhood affects maternal and child language skills [8], including less maternal speech and reduced sentence complexity [27, 28]. Studies of mothers that span the childbearing age range have identified that maternal depression, particularly postnatal depression, is associated with less responsive caregiving environments, reduced maternal language stimulation and poorer offspring cognitive and language development [29]. Similarly, exposure to maternal substance abuse during pregnancy and the postnatal period is associated with poorer offspring language outcomes and sub-optimal childrearing practices [30]. Higher levels of social support and consistent father involvement are associated with home environments that are more supportive of offspring language development [31].

In terms of child related factors, children who are female and show linguistic superiority in the early years are more likely to elicit rich conversation from their caregiver [32]. Language ability is reduced in children who are born very preterm (< 32 weeks’ gestational age) and with a very low birthweight (< 1500 g), where delays in language development extend into the preschool years [33]. Longer breastfeeding duration is also associated with better child cognitive outcomes at ages two and three [34]. The association between these risk and protective factors and the language outcomes of young mothers’ and children therefore requires examination.

The primary aim of our study was to investigate the effectiveness of the FNP in improving the language outcomes of young mothers and their children. We coded video-recordings of mothers interacting with their 24-month-old children for features of both maternal and child language production. We hypothesized that mothers and children in the FNP trial arm would produce more speech, and more complex speech, than mothers and children who received usual care. We also conducted an exploratory investigation of the family environment, maternal, and child related predictors of mothers’ and children’s language outcomes.

## Method

### Design

The Building Blocks trial was a randomised control trial (RCT) designed to investigate the effectiveness of the FNP programme in England. The Building Blocks trial took place within 18 sites across England, where local partnerships, including primary and secondary National Health Service (NHS) organisations and local authorities were established to provide FNP. Participants in the Building Blocks trial were allocated to receive either FNP (in addition to usually provided health and social care services) or usual care alone. Participants were randomly allocated to trial arm in a ratio of one-to-one, stratified by site and minimised by gestation (< 16 weeks vs. ≥ 16 weeks), smoking (yes vs. no), and preferred language of data collection (English *vs* non-English) with a probability of 0.80. Following baseline data collection prior to randomisation, follow-up data collection took place in late pregnancy, and at six, 12, 18 and 24 months postpartum. This study is a secondary analysis of data from the Building Blocks trial, whose sample size was based on four primary outcomes (2 maternal; 2 infant). Therefore, this exploratory study is not likely to be sufficiently powered. This trial is registered with ISRCTN, number ISRCTN23019866, 20/04/2009.

### Participants

**Fig. 1:**
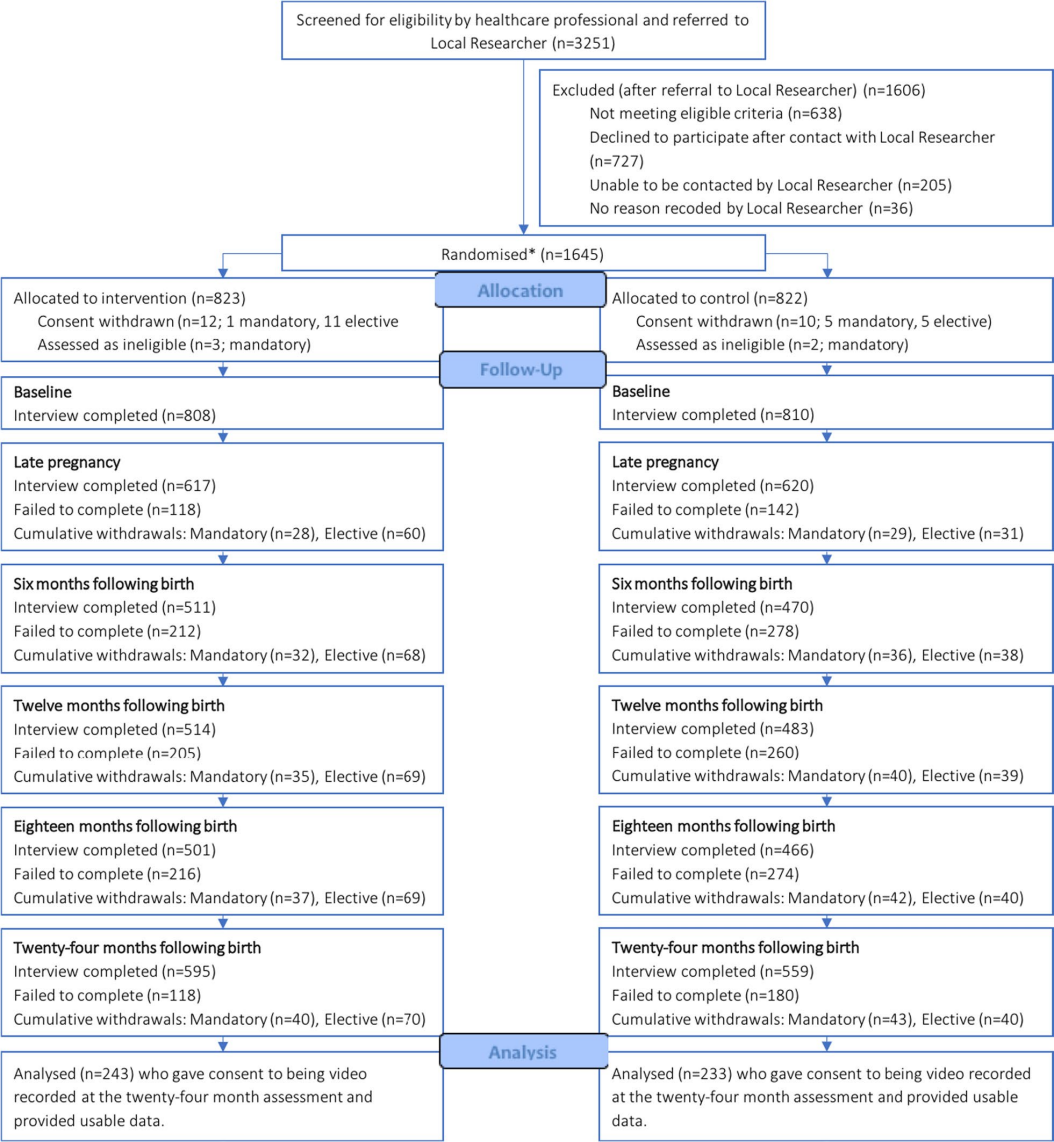
Flow diagram of progression to sample

The original trial included 1645 nulliparous women who at recruitment were 19 years or younger, of less than 25 weeks’ gestation, were living within an FNP catchment area, and able to provide consent and speak and understand English. Maternal mental health status and intentionality of the pregnancy was neither an FNP programme enrolment nor a trial eligibility criterion. The trial was undertaken at 18 sites across in England. Women were identified and approached via local maternity services and recruited usually at their home by locally based researchers. Participants were allocated to the FNP (*N* = 823) or usual care trial arms (*N* = 822). Of these participants, 1154 took part (mostly in person) in the data collection interview at the 24-month follow-up. The present study focuses on the subsample of 483 (41.9%) mothers and their children (237, 49.1% female) who consented to being video recorded during a mother–child interaction task at the 24-month assessment. Of the N = 483 eligible families, five were excluded from the analysis as the parent–child interaction task was conducted in a language other than English. For two families, there were technical errors at the point of data collection and we were unable to score the data. These participants formed the sample for the BABBLE sub-study (see Fig. 1).

### Procedure

*FNP intervention* FNP is an evidence-based nurse-led intensive home visiting programme for women expecting their first baby. FNP was originally developed in the USA (University of Colorado, Denver) as the Nurse Family Partnership [17], and is designed to improve perinatal, offspring health, developmental outcomes and parental economic self-sufficiency. The programme is based on three theoretical approaches: attachment theory [35]; ecological theory [36]; and self-efficacy theory [37]. In the adaptation of the programme, trained Family Nurses conduct home visits from early pregnancy until the child reaches 24-months of age. Although the number of visits is determined by individual need, families can receive a maximum of 64 scheduled visits: 14 during pregnancy, 28 during infancy (0–12 months postpartum) and 22 during toddlerhood (13–24 months postpartum). In the main trial cohort, the median number of valid visits reported by family nurses as being received by FNP clients was: pregnancy 10 (8–12), infancy 19 (14.5–22) and toddlerhood 13 (8–16). The mean (SD) visit duration varied by delivery phase (pregnancy: 79.14 min (13.78), infancy: 73.17 (11.61), toddlerhood: 74.75 (13.50) which exceeded the programme target of 60 min. Family nurses cover content including personal and environmental health, life course development, maternal role, family and friends and access to health and Social Services. Several programme elements are considered especially relevant to either parent–child interaction or child language development. These include the use of interventions delivered during home visits based on PIPE (Parents in Partnership Education), assessments of parent responsiveness (Nursing Child Assessment Satellite Training, NCAST) and a strengths-based approach used by family nurses within the therapeutic relationship involving modelling of positive communication styles. Family nurses also deliver the universally offered Healthy Child Programme (HCP).

*Usual care* Participants in the usual care arm received care from their local maternity services, as well as their postnatal midwifery care and support from existing child health services available locally, including an allocated Health Visitor.

*Data collection* Routine data such as antenatal, birth and neonatal data were collected from maternity records. Secondary care data (e.g., emergency admissions) were collected via the NHS Health and Social Care Information Centre, now NHS Digital. Primary care data were collected directly from primary care records for a proportion of participants. At baseline (< 25 weeks’ gestation) and at 24 months’ postpartum, computer-assisted personal interviews were conducted by researchers. Computer-assisted telephone interviews were conducted at late pregnancy and six, 12 and 18 months postpartum by office-based researchers. Participants were not blind to the intervention. However, at the baseline, late pregnancy, six, 12 and 18 month assessments, data collection was completed by field researchers blind to the trial arm. While 24-month interviews were completed by researchers not blinded to trial arm, they occurred independent of service delivery.

For those families who provided additional consent at the 24-month assessment, mother–child dyads were video recorded as they interacted during a 3-min free play session. Each mother–child dyad was provided with a standardized set of age-appropriate toys (stacking cups, bells, a stuffed Winnie the Pooh/Tigger and a wind-up car). Mother–child dyads were asked to play however they liked and could also use their own toys.

*Transcriptions of speech* All meaningful speech by mothers and children was transcribed verbatim from the audio-visual records by trained research assistants who were blind to trial arm. All speech was divided into utterances, defined as speech bounded by grammatical closure, a transition in speaker, or by a pause or change in intonational pattern [38]. Transcripts included all mother and child meaningful clear speech referring to entities, properties or events. Transcript agreement was established for 24 (5%) of cases with agreement at 91.4% for maternal speech, and 90.8% for child speech. Of those who provided audio-visual data, seven dyads were excluded (five did not take place in English and two had technical errors at the point of data collection), resulting in a final subsample of 476/483 (98.6%; FNP n = 243, usual care n = 233) mother–child dyads.

### Measures

*Mother and child language* Frequently used measures of maternal and child language production were coded in the current study [27, 28, 38], by coders blind to the trial arm. Second research assistants who were also blind to the trial arm coded random sub-samples of 71/476 (15%) cases to establish reliability for coding of mother and child language.

*Mean length of utterance* Sentence complexity was calculated in line with Brown’s [39] classic recommendations for calculating *mean length of utterance* (MLU) in morphemes: (1) only full, clear words were coded; (2) all exact utterance repetitions were included, but not repeated efforts at a single word; (3) fillers such as “mm,” and “oh,” were excluded; (4) compound words, proper names, and ritualised reduplications were coded as single morphemes (e.g., “choo-choo,” “night-night” “PeppaPig”); (5) irregular verbs (e.g., “ran”) were coded as single morphemes; (6) diminutives (e.g., “doggy”, “mummy”) were coded as single morphemes; and (7) all auxiliaries (e.g., “is,” “have,” “can,”) and catenatives (e.g., “gonna,” “wanna,” “hafta”) were counted as single morphemes, as were all morphemes with inflections (i.e., possessive [-s], plural [-s], third person singular [-s], regular past [-s], and progressive [-ing]). Inter-rater reliability for mother MLU (*intraclass correlation [ICC]* = 0.99) and child MLU (*ICC* = 0.96) were both excellent.

*Upper bound MLU* Both mothers’ and children’s longest utterance in morphemes was recorded as their *upper bound*. Excellent reliability was established for both mother *ICC* = 0.96 and child (*ICC* = 0.83) upper bound MLU.

*Tokens* Mother and child utterances were coded for number of words (tokens). All meaningful speech (therefore excluding fillers such as “mm” and “oh”) were counted. Excellent reliability was established for mother and child tokens (*ICCs* = 0.95 and 0.99 respectively).

*Types* Mother and child utterances were also coded for the number of different root words they produced (*types)* as a measure of lexical diversity. All inflected forms of words were coded as the same type (e.g., “run,” “running” = 1 type; “eye, “eyes = 1 type), as were irregular verbs (e.g., “run,” “ran” = 1 type). Words with different derivational morphology were treated as different types (e.g., “love,” “lovely” = 2 types). Words were also coded as 1 word type if they were a variation of a proper name or a proper name containing more than one word (e.g., “Lily,” “Lil,” = 1 type; “mummy”, “mumma”; “mum,” = 1 type “WinnieThePooh” = 1 type; [28]). Excellent reliability was established for mother types (*ICC* = 0.99) and child types (*ICC* = 0.99).

*Early language milestones scale* Children’s language ability was reported the Early Language Milestones (ELM) scale at the 24-month assessment, which is based on maternal reports with some elicitation of child behaviour (ELM; [40]). The ELM has been established as a sensitive indicator of children’s early language development in high-risk populations [40]. Children’s performance was calculated using the point scoring method to yield percentile values for overall language ability, and for the Auditory Expressive and Receptive subscales.

*Sociodemographic characteristics* Variables included: (a) maternal age at recruitment; (b) language spoken in the home (English or English and other); (c) relationship status with the focal child’s father at the 24-month assessment; (d) not in education, employment or training (NEET) at the 24-month assessment; and (e) number of people living in the home at the time of the 24-month assessment. At recruitment, an overall measure of deprivation was recorded using the Index of Multiple Deprivation (IMD; [41]), a measure comprising seven weighted factors that yield a score between 0 and 100 (least to most deprived). These include: (1) income; (2) employment; (3) health deprivation and disability; (4) education, skills and training; (5) barriers to housing and services; (6) crime and disorder; and (7) living environment.

*Maternal psychological distress* Mothers’ psychological distress at 24 months was assessed using the Kessler Psychological Distress Scale, a 10-item screening scale that discriminates with precision DSM-IV cases from non-cases [42]. Mothers were asked to report how they had been feeling during the last 30 days, for example, “How often did you feel tired out for no good reason?” and “How often did you feel hopeless?” Mothers’ responses were scored between 10 and 50 (higher scores indicated more distress). Reliability of the Kessler Psychological Distress Scale was assessed using Cronbach’s alpha, and was found to be excellent at 0.91.

*Postnatal depression* Postnatal depression (PND) was assessed at the six-month follow-up assessment using the Edinburgh Postnatal Depression Scale (EPDS; [43]). In this 10-item scale, mothers were asked to report how they had felt in the last seven days on items such as “I have felt scared or panicky for no very good reason” and “I have felt sad or miserable” on a scale of 0 = no, not at all, 1 = no, not much, 2 = yes, sometimes, or 3 = yes, quite a lot. Where necessary, items were reverse scored. Mothers’ symptoms of PND were scored between 0 and 30 (higher scores indicated more symptoms). Reliability of the scale was found to be good (Cronbach’s alpha = 0.86).

*Social support* Mothers’ perception of her social support and networks (MOS survey; [44]) was assessed at 24-months. Mothers selected one of five possible responses, 0 = never, 1 = seldom, 2 = sometimes, 3 = almost always, and 4 = always on 19 items such as “If you need it, how often can you depend on somebody for help?” Responses were scaled to a score between 0 and 100; higher scores indicated higher social support. Reliability of the scale was found to be excellent (Cronbach’s alpha = 0.96).

*Substance abuse* The CRAFFT substance and abuse screening test [45] was used to assess maternal problems with alcohol and drug use at 24 months. Mothers answered 0 = no or 1 = yes to 6 items, for example, “Do you ever forget things you did while using alcohol or drugs?” and “Have you ever gotten into trouble while you were using alcohol or drugs?” Mothers could score between 0 and 6, where higher CRAFFT scores indicated greater problems related to drug and alcohol use. Reliability of the CRAFFT scale was poor (Cronbach’s alpha = 0.56).

*Pregnancy, birth, and parenting behaviour* Childbirth data was collected from maternity records, and included *gestation at birth* (in weeks), *birthweight* (in grams). Information about maternal *antenatal smoking* was collected at baseline and in pregnancy. The final antenatal smoking variable used was a calibrated measure of cigarettes smoked per day during late pregnancy using a combination of urinary cotinine results (collected at late pregnancy) and self-reported number of cigarettes smoked per day (based on three days prior to the interview, time of last cigarette, and hours since last cigarette). Mothers’ *duration of breastfeeding* (in days) was reported at six months, and updated at 12, 18, and 24 months.

### Data analysis

Data preparation and analysis was conducted using SPSS version 20 and Stata version 13. Trial arm differences in children’s productive language were tested adjusting for minimisation variables: gestation (< 16 weeks *vs* > 16 weeks); smoking (yes vs. no); and language (English vs. non-English). Models were further adjusted using multilevel modelling to adjust for site. Child ELM scores were analysed using linear regression. Mothers’ MLU, types and tokens were treated as continuous data and analysed using linear regression. All assumptions for linear regression were met. Normality of residuals were assessed by inspection of histograms and Normal P-P Plots, and independence of observations using the Durbin-Watson Test. For children, due to high counts of single-morpheme utterances resulting in skewed data, child MLU was categorised as: 0 = 0 counts, 1 = single-morpheme use, and 2 ≥ 1 morphemes and therefore analysed using ordinal regression. Mothers’ upper bound MLU and children’s types and tokens were count data, and were analysed using poisson/negative binomial regression as appropriate. Linear regression models are presented as adjusted mean differences in the FNP group minus the usual care group. Ordinal regression models are presented as odds ratios (*OR*s), and poisson/negative binomial regression models are presented as incident rate ratios (*IRR*s).

Predictors of the primary feature of mother and child observed language (MLU in morphemes) were first assessed on the univariable level, using linear and ordinal regressions, respectively. Identified sample-wide predictors that reached 10% significance level in the bivariate tests were entered in a multivariable analysis, with associations reaching *p* < 0.05 reported as significant in multivariable models. Linear and ordinal regressions are presented as mean differences and odds ratios, respectively. Analyses of predictor variables in all the models showed no collinearity (variance inflation factor < 10, tolerance > 0.20; Myers, 1990).

## Results

### Description of sample

The baseline sociodemographic characteristics of the families who participated and consented to filming at the 24 month assessment compared to the rest of the Building Blocks sample is detailed the supplementary material. Participants in the FNP trial arm of the BABBLE sub-study received more FNP visits than those who did not take part in the sub-study. The BABBLE sample was also imbalanced in terms of ethnicity and more spoke only English in the home. Of the 483 mother–child dyads who took part in BABBLE, 246 dyads were allocated to FNP (50.9%), and 237 to usual care (49.1%). The BABBLE subsample was balanced according to baseline sociodemographic characteristics between the FNP and usual care groups.

### Sample-wide description of mother and child language

All mothers in the BABBLE sample made at least one utterance to their child during free-play, with the majority of children (*N* = 432, 90.8%) making at least one utterance. Of those who made at least one utterance, 129 (29.9%) produced only single-morpheme utterances and 303 (63.7%) produced multi-morpheme utterances. Table 1 shows the descriptive data for the BABBLE sample for mother and child productive language: MLU in morphemes, upper bound MLU, tokens, and types. Mothers’ reports of child language at 24 months are also presented in Table 1. Measures of children’s observed language and mothers’ reports of child language ability on the ELM were significantly associated (*r* = 0.45 s-0.50 for all coded outcomes, all *p* < 0.01).

**Table 1:** Descriptive data for measures of maternal and child language by trial arm

		Mean	SD	Range	Median	IQR
*Mother*						
**Observational data**	**MLU**					
	Full sample	3.01	0.57	1.44–4.75	3.00	2.64–3.37
	FNP	3.06	0.52	1.68–4.66	3.03	2.72–3.42
	Usual care	2.96	0.61	1.44–4.75	2.95	2.55–3.34
	**Upper bound MLU**					
	Full sample	8.46	2.03	3.00–16.00	8.00	7.00–10.00
	FNP	8.62	1.89	3.00–15.00	8.00	7.00–10.00
	Usual care	8.30	2.15	3.00–16.00	8.00	7.00–10.00
	**Tokens**					
	Full sample	165.03	65.40	15.00–403.00	162.00	118.00–208.00
	FNP	168.39	60.93	19.00–403.00	167.00	128.00–205.00
	Usual care	161.52	69.71	15.00–383.00	154.00	111.00–213.00
	**Types**					
	Full sample	59.82	17.82	9.00–116	59.00	48.00–72.00
	FNP	61.31	16.93	10.00–116.00	61.00	51.00–72.00
	Usual care	58.37	18.62	9.00–113.00	57.00	46.50–71.00
*Child*						
**Observational data**	**MLU**					
	Full sample	1.22	0.54	0.00–3.56	1.19	1.00–1.50
	FNP	1.24	0.53	0.00–3.56	1.20	1.00–1.50
	Usual care	1.20	0.56	0.00–3.08	1.19	1.00–1.50
	**Upper bound MLU**					
	Full sample	2.30	1.66	0.00–10.00	2.00	1.00–3.00
	FNP	2.32	1.73	0.00–10.00	2.00	1.00–3.00
	Usual care	2.29	1.60	0.00–9.00	2.00	1.00–3.00
	**Tokens**					
	Full sample	14.49	14.69	0.00–142.00	11.00	3.00–21.00
	FNP	14.27	15.99	0.00–142.00	9.00	4.00–19.00
	Usual care	14.73	13.24	0.00–68.00	13.00	3.00–22.00
	**Types**					
	Full sample	7.86	6.97	0.00–52.00	6.00	2.00–12.00
	FNP	7.72	7.15	0.00–52.00	6.00	3.00–11.00
	Usual care	7.99	6.80	0.00–33.00	7.00	2.00–7.00
**Assessment Data**	**ELM**^+^					
	Full sample	60.77	31.85	0.00–99.00	65.00	32.50–95.00
	FNP	63.32	31.48	0.00–99.00	75.00	35.00–95.00
	Usual care	58.11	32.10	0.00–99.00	62.50	30.00–90.00
	**ELM AE**^+^					
	Full sample	54.10	32.33	2.00–98.00	50.00	25.00–90.00
	FNP	57.07	31.85	2.00–98.00	60.00	30.00–90.00
	Usual care	51.02	32.62	2.00–98.00	50.00	20.00–80.00
	**ELM AR**^+^					
	Full sample	65.86	29.17	2.00–98.00	75.00	45.00–90.00
	FNP	68.00	28.47	2.00–98.00	80.00	45.00–90.00
	Usual care	63.63	29.77	2.00–98.00	75.00	41.25–90.00

Full sample* N* = 476, ^+^*n* = 457. FNP *n* = 243*,* ^+^*n* = 233. Usual care *n* = 233, ^+^*n* = 224

*SD* standard deviation. *IQR* interquartile range. *MLU* mean length of utterance in morphemes. *ELM* Early Language Milestones Scale. *ELM AE* Early Language Milestones Auditory Expressive Subscale. *ELM AR* Early Language Milestones Auditory Receptive Subscale

### Trial arm differences in mother and child language

Table 1 shows the descriptive data for observed mother language measures and reported and observed child language measures by trial arm.

*Trial arm differences in mother language* Significant differences were detected between the FNP and usual care groups in mother MLU (Table 1), where mothers in the FNP group produced a higher MLU within mother–child interaction than those who received usual care, adjusted difference in means (adjusted by minimisation variables and by site, linear regression) = 0.10, *p* < 0.05, 95% *CI* (0.004–0.20), *d* = 0.18. No differences were detected between the trial arms for mother upper bound MLU, adjusted IRR (Poisson regression) = 1.03, *p* = 0.21, 95% *CI* (0.97–1.10), *d* = 0.02. Similarly, no differences were detected between trial arms in terms of mothers’ tokens, adjusted difference in means (linear regression) = 6.78, *p* = 0.25, 95% *CI* (− 4.95 to 18.52), *d* = 0.10, or mothers’ types, adjusted difference in means (linear regression) = 2.84, *p* = 0.08, 95% *CI* (− 0.36 to 6.03), *d* = 0.16.

*Trial arm differences in child language* Although the differences did not reach statistical significance, analysis of the ELM total score in the BABBLE subsample showed similar trial arm differences to those found in the full-sample [25], adjusted difference in means (adjusted by minimisation variables and by site, linear regression) = 4.01, 95% *CI* (− 1.57 to 9.58), *p* = 0.15, Cohen’s *d* = 0.16 (*d* = 0.14 in full sample). No differences were detected between the trial arms for child MLU, adjusted *OR* (ordinal regression) = 1.07, 95% *CI* (0.74–1.55), *p* = 0.72, *d* = 0.04; child upper bound MLU, adjusted *IRR* (negative binomial regression) = 1.01, 95% *CI* (0.89–1.15), *p* = 0.85, *d* = 0.01; tokens adjusted *IRR* (negative binomial regression) = 0.97, 95% *CI* (0.80–1.17), *p* = 0.74, *d* = 0.02; and types adjusted *IRR* (negative binomial regression) = 0.97, 95% *CI* (0.82–1.14), *p* = 0.68, *d* = 0.02).

### Predictors of mother and child language

Descriptive statistics for family environment, maternal, and child characteristics are presented in Table 2. These variables were investigated as predictors of the primary mother and child observed language outcomes (MLU).

**Table 2:** Descriptive data for predictors of maternal and child language production

Sociodemographic characteristics	BABBLE observation sample (*N* = 476)
*Maternal age at recruitment (years)*	
Mean (SD)	17.91 (1.22)
Range	13.82–19.98
*Language spoken in the home N (%)*	
English	467 (98.1)
Other	9 (1.9)
*NEET status at 24 months N (%)*	
Yes	307 (64.5)
No	169 (35.5)
*Relationship with child’s father at 24 months*^*+*^* N (%)*	
Married	15 (3.2)
Separated/divorced	25 (5.3)
Closely involved/boyfriend	208 (43.7)
Just friends	91 (19.1)
Not in any relationship	136 (28.6)
*Number of people living in the home at 24 months*	
Mean (SD)	1.03 (1.31)
Range	0–7
*IMD score at baseline*	
Mean (SD)	38.52 (17.96)
Range	3.15–82.00
*Maternal health and wellbeing*	
Psychological distress at 24 months	
Mean (SD)	17.10 (1.27)
Range	10–43
Postnatal depression at 6 months	
Mean (SD)	6.71 (5.05)
Range	0–24
Social support at 24 months	
Mean (SD)	84.41 (17.74)
Range	11.84–100
Problem alcohol and drug use at 24 months	
Mean (SD)	0.36 (0.80)
Range	0–5
*Pregnancy, birth, and parenting behaviour*	
Gestation at birth (weeks)	
Mean (SD)	39.31 (1.89)
Range	27–42
Birthweight (grams)	
Mean (SD)	3284.53 (534.11)
Range	993–4700
Antenatal smoking (number of cigarettes) in 2nd & 3rd trimester	
Mean (SD)	4.61 (5.94)
Range	0–24.07
Duration breastfeeding (days)	
Mean (SD)	20.81 (44.64)
Range	0–194

^+^Only 1 in sample was divorced, so merged with ‘separated’; Language spoken in the home was not examined as a predictor variable due to the majority having English language only

*SD* standard deviation; *NEET* not in education, employment or training; *IMD* index of multiple deprivation

Higher IMD score indicates more deprivation. Mean IMD score for England in 2010 was 21.67 [41]

*Predictors of mother MLU* Bivariate associations were first investigated using a series of linear regressions (see Additional file 1: Table S2 for all analyses). Mothers not being in education, training or employment and those with minimal social support had lower maternal MLU scores, *ß* = − 0.199, 95% *CI* (− 0.304 to -0.093) and *ß* = 0.003, 95% *CI* (0.000–0.006) respectively. In contrast, higher ratings of problematic drug use and a greater duration of breastfeeding was associated with higher maternal MLU, *ß* = 0.056, 95% *CI* (− 0.008 to 0.120) and *ß* = 0.002, 95% *CI* (0.001–0.004) respectively. In terms of child factors, higher child MLU scores and the child being female were associated with higher maternal MLU, *ß* = 0.240, 95% *CI* (0.148–0.331) and *ß* = 0.109, 95% *CI* (0.008–0.211) respectively (all *p*s < 0.10) These predictors of mother MLU were entered together into a multivariate linear regression (Table 3). In the final model, mothers’ education, employment or training (NEET) status and her report of substance abuse at 24 months remained significant predictors of mothers’ MLU. The duration of breastfeeding and child MLU also remained significant in the model (all *p*s < 0.05, Table 3).

**Table 3:** Multivariatble analysis of predictors of maternal MLU (Model 1) and child MLU (Model 2)

Model 1	*B*	95% *CI*	*p*-value
*NEET status at 24 months*			
No	Reference		
Yes	− 0.161	− 0.264 to − 0.058	.002
Social support at 24 months	0.003	0.000–0.005	.066
Child MLU	0.200	0.107–0.294	.000
*Sex of baby*			
Male	Reference		
Female	0.059	− 0.042 to 0.159	.253
Substance abuse at 24 months	0.062	0.000–0.124	.050

Model 2	Odds ratio	95% *CI*	*p*-value
*NEET status at 24 months*			
No	Reference		
Yes	0.68	0.45–1.03	.071
Mother psychological distress at 24 months	0.98	0.96–1.01	.313
Mother MLU	1.61	1.13–2.29	.007
*Sex of baby*			
Male	Reference		
Female	2.36	1.59–3.49	.000
Number of weeks gestation	1.08	0.95–1.23	.224
Birth weight	1.00	1.00–1.01	.705

Model 1, predictors of maternal MLU. *N* = 472. Multivariable linear regression. Number of days breastfeeding was entered in a separate regression model with reduced sample who had data available (*N* = 339) and was also a significant predictor of mothers’ MLU, Odds ratio = .002, 95% *CI* (0.001–0.003), *p* = .003. Model 2, predictors of child MLU. *N* = 475. Multivariate ordinal regression

*NEET* not in education, employment or training; *MLU* mean length of utterance in morphemes

*Predictors of child MLU* Bivariate associations were first tested using a series of ordinal regressions (see Additional file 1: Table S3 for all analyses). Mothers not being in education, employment or training (NEET) was associated with lower child MLU, *OR* = 0.60, 95% *CI* (0.40–0.90). Similarly, higher maternal psychological distress was also associated with lower child MLU, *OR* = 0.97, 95% *CI* (0.95–1.00). Higher maternal MLU was associated with higher child MLU *OR* = 1.86, 95% *CI* (1.32–2.63). Children who were female, *OR* = 2.53, 95% *CI* (1.73–3.70), who were born at a higher number of weeks gestation, *OR* = 1.13, 95% *CI* (1.03–1.24), and higher birth weight, *OR* = 1.00, 95% *CI* (1.00–1.01), had higher MLU scores (all *p*s < 0.10). These variables were entered together into an ordinal regression model, where higher mother MLU and the child being female significantly predicted higher child MLU at 24 months (all *p*s < 0.05, Table 3).

## Discussion

In the context of this pragmatic randomised controlled trial we examined the impact of the FNP home-visiting programme on mothers’ and toddler’s language production during a parent–child interaction task. We detected a small but significant difference between the FNP and usual care groups in mothers’ MLU, where mothers in the FNP group showed higher MLU than the mothers who received usual care alone. No differences were detected between groups in other coded features of maternal or child language. Our findings suggest that FNP has a small, but positive impact on the language environment that mothers provide for their children. Given that enriched parent–child verbal engagement strengthens children’s processing and acquisition of language [28], the finding that FNP increased maternal MLU within a very brief observation of mother–child interaction is promising. Although such differences may be modest when considering the impact of highly-targeted interventions, for programmes that are rolled out at scale to larger populations of first-time mothers the population benefit may have important policy implications, especially if aligned to other programme benefits.

In contrast to our hypotheses, we found no statistically significant differences between the FNP and usual care control group for the language children produced during the parent–child interaction task. On the maternally reported ELM scale we observed almost identical differences in the current study to those found in the original trial—with the magnitude of this difference classified as a small effect size [25]. However, in contrast to the larger Building Blocks sample, the between group difference on the ELM was not statistically significant in the smaller BABBLE sub-sample. Measures of children’s observed language and mothers’ reports of children’s language ability were moderately correlated. This finding suggests that maternal reports of offspring language development on the ELM are a reasonably accurate reflection of children’s language ability. Nevertheless, the small benefits of FNP for children’s language development were not detectable during the brief observation of parent–child interaction conducted in the current study.

Our findings extend past research by examining mothers’ as well as children’s language outcomes following a home-visiting intervention [6, 11, 12, 15, 19]. To improve children’s language outcomes home-visiting programmes need firstly to enhance the language environment provided by parents [46, 47]. By embedding a parent–child interaction task within a large-scale pragmatic RCT we micro-analysed maternal language. Research shows that the language environment provided by parents is inextricably linked to children’s language development, which in turn predicts school readiness and subsequent behavioural and socio-emotional adjustment [8–10]. Increasing and enhancing parents’ interactions with their young children is an important component of home visiting programmes. Only the longer-term follow-up of the BABBLE sample will determine whether the impact of FNP on mother’s language interactions observed in the current study leads to improved offspring outcomes. Recent evidence from a data linkage study indicates small but positive effects of FNP on development (school readiness) and educational outcomes when children were approximately 7-years-old [48].

Identifying the mechanisms through which early intervention programmes improve children’s outcomes remains a challenge [49]. Our findings indicate that in addition to standardised interview, questionnaire and developmental assessments, future evaluations of home-visiting programmes would benefit from conducting observational assessments of parent–child interactions. Few evaluations of home-visiting programmes have conducted in-depth observational assessments of parental behaviour [15, 16]. In-depth assessments of specific domains of parental behaviour could help elucidate the processes that mediate beneficial programme effects. One such focus could be maternal responsiveness to child cues, which is robustly associated with child language development [50]. Other potential mechanisms that underpin the effects of early intervention programmes are also worthy of exploration, including processes within parents *(e.g. increased parental self-efficacy and reductions in barriers to effective parenting)*, processes within children *(e.g. reductions in early problem behaviours that persist over-time and/or increased resilience and self-regulation),* and improvements in parents and children’s transactions with their environmental contexts [49].

Our sample wide analyses of the predictors of maternal MLU showed that mothers who breastfed their babies for longer had higher MLU scores, whereas those not in employment, education or training had lower MLU scores. Increased breastfeeding duration could be a marker for other maternal behaviours associated with positive maternal-child outcomes. For example, breastfeeding duration is associated with greater maternal sensitive responsiveness which in turn is associated with more positive parent–child outcomes [51]. Contrary to hypotheses, mothers with a greater risk of problems associated with alcohol or drug use had higher MLU scores. Why this is, remains unclear. The substance and abuse screening test used in the current study captures the risk of impairment associated with alcohol/drug use rather than the frequency or severity of abuse. Future studies examining the relationship between maternal substance abuse and mother–child language production would benefit from using more comprehensive measures of maternal alcohol and illegal drug use.

The sample wide predictors of children’s MLU also showed that female offspring had higher MLU scores, whereas mothers not in employment, education or training had offspring with lower scores. Mothers with a higher MLU score also had children with a higher MLU score and vice versa. Similar correlates of mothers’ and children’s language production have been identified in other samples of children and their mothers who span the childbearing age range [32]. Furthermore, our findings highlight that young mothers not in employment, education or training represent a sub-group of mother–child dyads who are at particular risk of poorer language outcomes and could benefit from additional targeted intervention.

## Limitations and future directions

Compared to the families in the original sample who did not agree to be video-recorded, the families observed in the present sub-sample had more FNP home visits, more antenatal check-ups, and the sample was not representative on some sociodemographic variables. As such, differences in these variables could have resulted in the sub-sample not being representative of the original sample, if the higher dosage was driving some of our findings. Additionally, the observations of mother–child interaction were restricted to a 3-min free-play task, and therefore the speech samples available for analysis were limited. While MLU is considered a reliable and valid index of language acquisition [52], historically, MLU is typically calculated on the basis of 50 to 100 utterances, however, studies have demonstrated that short samples of speech (e.g., 1- to 3-min samples yielding as few as 12 utterances) show consistency with longer samples [53]. Similarly short observations have been used to successfully measure maternal linguistic input and mother–child interactions, e.g., [54, 55]. Sample size invariably results in a trade-off with length of observations. Although language production and quality of interaction are typically studied in concentrated time-periods, the use of a more naturalistic observation of mother–child dyads within the home may give a more accurate reflection of children’s daily conversational environments.

## Conclusions

This study furthers the evidence base for home-visiting programmes by taking an observational approach to study both mother and child language outcomes. Within a brief mother–child interaction task, we detected that FNP had a small but significant impact on young mothers’ speech to their toddlers. This finding supports the delivery of FNP as enriched parent–child verbal engagement promotes children’s language development. Although there was no detectable impact on children’s observed language production within the mother–child interaction task, our findings are encouraging given that intervention effects on positive child outcomes can be mediated by improvements in the quality of mother–child interactions [7]. As such, evidence for benefit of FNP on children’s language outcomes may become more apparent later in childhood. Longer-term follow-up studies of home-visiting programmes are needed to determine whether early indications of positive benefits translate into enduring educational, occupational and health related outcomes for women and children.

## Supplementary Information

**Additional file 1**.** Supplementary Table S1**. Baseline sociodemographic characteristics of BABBLE sample and non-BABBLE sample.** Supplementary Table S2**. Bivariate analyses of family environment, maternal and child predictors of maternal MLU.** Supplementary Table S3**. Bivariate analyses of family environment, maternal, and child predictors of children’s MLU.

## Data Availability

The datasets generated and/or analysed during the current study are not publicly available but are available from the corresponding author on reasonable request.

## References

[1] Moffitt TE, Team ERS (2002). Teen-aged mothers in contemporary Britain. J Child Psychol Psychiatry.

[2] Keown LJ, Woodward LJ, Field J (2001). Language development of pre-school children born to teenage mothers. Infant Child Dev Int J Res Pract.

[3] Oxford M, Spieker S (2006). Preschool language development among children of adolescent mothers. J Appl Dev Psychol.

[4] Jaffee S, Caspi A, Moffitt TE, Belsky J, Silva P (2001). Why are children born to teen mothers at risk for adverse outcomes in young adulthood? Results from a 20-year longitudinal study. Dev Psychopathol.

[5] Shaw M, Lawlor DA, Najman JM (2006). Teenage children of teenage mothers: psychological, behavioural and health outcomes from an Australian prospective longitudinal study. Soc Sci Med.

[6] Olds DL, Robinson J, O’Brien R, Luckey DW, Pettitt LM, Henderson CR (2002). Home visiting by paraprofessionals and by nurses: a randomized, controlled trial. Pediatrics.

[7] Baudry C, Tarabulsy GM, Atkinson L, Pearson J, St-Pierre A (2017). Intervention with adolescent mother–child dyads and cognitive development in early childhood: a meta-analysis. Prev Sci.

[8] Hart B, Risley TR (1995). Meaningful differences in the everyday experience of young American children.

[9] Zauche LH, Thul TA, Mahoney AED, Stapel-Wax JL (2016). Influence of language nutrition on children’s language and cognitive development: an integrated review. Early Child Res Q.

[10] Forrest CL, Gibson JL, Halligan SL, St Clair MC (2018). A longitudinal analysis of early language difficulty and peer problems on later emotional difficulties in adolescence: evidence from the Millennium Cohort Study. Autism Dev Lang Impair.

[11] Aracena M, Krause M, Pérez C, Méndez MJ, Salvatierra L, Soto M (2009). A cost-effectiveness evaluation of a home visit program for adolescent mothers. J Health Psychol.

[12] Olds DL, Kitzman H, Cole R, Robinson J, Sidora K, Luckey DW (2004). Effects of nurse home-visiting on maternal life course and child development: age 6 follow-up results of a randomized trial. Pediatrics.

[13] Schwartz M, Verschik A (2013). Achieving success in family language policy: Parents, children and educators in interaction. Successful family language policy.

[14] King TM, Rosenberg LA, Fuddy L, McFarlane E, Sia C, Duggan AK (2005). Prevalence and early identification of language delays among at-risk three year olds. J Dev Behav Pediatr.

[15] Nievar MA, Van Egeren LA, Pollard S (2010). A meta-analysis of home visiting programs: Moderators of improvements in maternal behavior. Infant Ment Health J.

[16] Tarabulsy GM, Moran G, Pederson DR, Provost M, Larose S. Adolescent motherhood, maternal sensitivity and early infant development. In: Davis DW, Logdson C, editors, Maternal sensitivity: a critical review for practitioners. Haupauge, NY: Nova.

[17] Olds DL, Henderson CR, Kitzman H (1994). Does prenatal and infancy nurse home visitation have enduring effects on qualities of parental caregiving and child health at 25 to 50 months of life?. Pediatrics.

[18] Kitzman H, Olds DL, Henderson CR, Hanks C, Cole R, Tatelbaum R (1997). Effect of prenatal and infancy home visitation by nurses on pregnancy outcomes, childhood injuries, and repeated childbearing: a randomized controlled trial. JAMA.

[19] Olds DL, Robinson J, Pettitt L, Luckey DW, Holmberg J, Ng RK (2004). Effects of home visits by paraprofessionals and by nurses: age 4 follow-up results of a randomized trial. Pediatrics.

[20] Olds DL, Holmberg JR, Donelan-McCall N, Luckey DW, Knudtson MD, Robinson J (2014). Effects of home visits by paraprofessionals and by nurses on children: follow-up of a randomized trial at ages 6 and 9 years. JAMA Pediatr.

[21] Evans RE, Craig P, Hoddinott P, Littlecott H, Moore L, Murphy S (2019). When and how do ‘effective’ interventions need to be adapted and/or re-evaluated in new contexts? The need for guidance.

[22] Sierau S, Dähne V, Brand T, Kurtz V, von Klitzing K, Jungmann T (2016). Effects of home visitation on maternal competencies, family environment, and child development: a randomized controlled trial. Prev Sci.

[23] Jack SM, Busser LD, Sheehan D, Gonzalez A, Zwygers EJ, MacMillan HL (2012). Adaptation and implementation of the Nurse-Family Partnership in Canada. Can J Public Health.

[24] Mejdoubi J, van den Heijkant SC, van Leerdam FJ, Crone M, Crijnen A, HiraSing RA (2014). Effects of nurse home visitation on cigarette smoking, pregnancy outcomes and breastfeeding: a randomized controlled trial. Midwifery.

[25] Robling M, Bekkers M-J, Bell K, Butler CC, Cannings-John R, Channon S (2016). Effectiveness of a nurse-led intensive home-visitation programme for first-time teenage mothers (Building Blocks): a pragmatic randomised controlled trial. Lancet.

[26] Barlow J, Barnes J, Sylva K, Fonagy P, Fearon P (2016). Questioning the outcome of the Building Blocks trial. Lancet.

[27] Hoff E (2003). The specificity of environmental influence: Socioeconomic status affects early vocabulary development via maternal speech. Child Dev.

[28] Huttenlocher J, Waterfall H, Vasilyeva M, Vevea J, Hedges LV (2010). Sources of variability in children’s language growth. Cogn Psychol.

[29] Stein A, Pearson RM, Goodman SH, Rapa E, Rahman A, McCallum M (2014). Effects of perinatal mental disorders on the fetus and child. Lancet.

[30] Viteri OA, Soto EE, Bahado-Singh RO, Christensen CW, Chauhan SP, Sibai BM (2015). Fetal anomalies and long-term effects associated with substance abuse in pregnancy: a literature review. Am J Perinatol.

[31] Pancsofar N, Vernon-Feagans L, Investigators FLP (2010). Fathers’ early contributions to children’s language development in families from low-income rural communities. Early Child Res Q.

[32] Barnett MA, Gustafsson H, Deng M, Mills-Koonce WR, Cox M (2012). Bidirectional associations among sensitive parenting, language development, and social competence. Infant Child Dev.

[33] Barre N, Morgan A, Doyle LW, Anderson PJ (2011). Language abilities in children who were very preterm and/or very low birth weight: a meta-analysis. J Pediatr.

[34] Bernard JY, De Agostini M, Forhan A, Alfaiate T, Bonet M, Champion V (2013). Breastfeeding duration and cognitive development at 2 and 3 years of age in the EDEN mother–child Cohort. J Pediatr.

[35] Bowlby J (1998). Attachment and loss.

[36] Bronfenbrenner U (1979). The ecology of human development: experiments by nature and design.

[37] Banciura A (1977). Self-efficacy: toward a unifying theory of behavior change. Psychol Rev.

[38] Suskind DL, Leffel KR, Graf E, Hernandez MW, Gunderson EA, Sapolich SG (2016). A parent-directed language intervention for children of low socioeconomic status: a randomized controlled pilot study. J Child Lang.

[39] Brown R (1973). Development of the first language in the human species. Am Psychol.

[40] Coplan J, Gleason JR, Ryan R, Burke MG, Williams ML (1982). Validation of an Early Language Milestone Scale in a high-risk population. Pediatrics.

[41] Wilkinson DL, Sniehotta FF, Michie S (2011). Targeting those in need: baseline data from the first English National Health Service (NHS) Health Trainer Service. Psychol Health Med.

[42] Kessler RC, Andrews G, Colpe LJ, Hiripi E, Mroczek DK, Normand S-L (2002). Short screening scales to monitor population prevalences and trends in non-specific psychological distress. Psychol Med.

[43] Cox JL, Holden JM, Sagovsky R (1987). Detection of postnatal depression: development of the 10-item Edinburgh Postnatal Depression Scale. Br J Psychiatry.

[44] Stewart AL, Hays RD, Ware JE (1988). The MOS short-form general health survey: reliability and validity in a patient population. Med Care.

[45] Knight JR, Sherritt L, Shrier LA, Harris SK, Chang G (2002). Validity of the CRAFFT substance abuse screening test among adolescent clinic patients. Arch Pediatr Adolesc Med.

[46] Landry SH, Zucker TA, Williams JM, Merz EC, Guttentag CL, Taylor HB (2017). Improving school readiness of high-risk preschoolers: combining high quality instructional strategies with responsive training for teachers and parents. Early Child Res Q.

[47] Landry SH, Smith KE, Swank PR, Guttentag C (2008). A responsive parenting intervention: the optimal timing across early childhood for impacting maternal behaviors and child outcomes. Dev Psychol.

[48] Robling M, Lugg-Widger FV, Cannings-John R, Angel L, Channon S, Fitzsimmons D (2022). Nurse-led home-visitation programme for first-time mothers in reducing maltreatment and improving child health and development (BB:2–6): longer-term outcomes from a randomised cohort using data linkage. BMJ Open.

[49] Sandler IN, Schoenfelder EN, Wolchik SA, MacKinnon DP (2011). Long-term impact of prevention programs to promote effective parenting: lasting effects but uncertain processes. Annu Rev Psychol.

[50] Madigan S, Prime H, Graham SA, Rodrigues M, Anderson N, Khoury J (2019). Parenting behavior and child language: a meta-analysis. Pediatrics.

[51] Tharner A, Luijk MP, Raat H, IJzendoorn MH, Bakermans-Kranenburg MJ, Moll HA (2012). Breastfeeding and its relation to maternal sensitivity and infant attachment. J Dev Behav Pediatr.

[52] Rice ML, Smolik F, Perpich D, Thompson T, Rytting N, Blossom M (2010). Mean length of utterance levels in 6-month intervals for children 3 to 9 years with and without language impairments. J Speech Lang Hear Res.

[53] Heilmann J, Nockerts A, Miller JF (2010). Language sampling: does the length of the transcript matter?. LHSS.

[54] Smith J, Levickis P, Goldfeld S, Kemp L, Conway L (2021). Maternal linguistic input and child language in a cohort at risk of experiencing social adversity. Lang Learn Dev.

[55] Lloyd CA, Masur EF (2014). Infant behaviors influence mothers’ provision of responsive and directive behaviors. Infant Behav Dev.

