# Supplementary material for: The impact of a specialist home-visiting intervention on the language outcomes of young mothers and their children: a pragmatic randomised controlled trial
Source: BMC Psychol. 2022 Sep 23;10:224. doi: 10.1186/s40359-022-00926-1 (PMC9508755; doi:10.1186/s40359-022-00926-1)
Supplement: Supplementary file 1 — Additional file 1. Supplementary Table S1. Baseline sociodemographic characteristics of BABBLE sample and non-BABBLE sample. Supplementary Table S2. Bivariate analyses of family environment, maternal and child predictors of maternal MLU. Supplementary Table S3. Bivariate analyses of family environment, maternal, and child predictors of children’s MLU. [file 40359_2022_926_MOESM1_ESM.docx]

**Supplementary Material**

Supplementary Table S1. *Baseline sociodemographic characteristics of BABBLE sample and non-BABBLE sample.*

|  | Total sample  (*N* = 1618) | BABBLE sample  (*N* = 483) | Non-BABBLE sample  (*N* = 1135) |
| --- | --- | --- | --- |
| Assigned to FNP *N* (%) | 808 (49.9) | 246 (50.9) | 562 (49.5) |
| Percentage of FNP visits across all phases (% of expected) (mean, *SD*)* | 56.14 *(29.29)* | 66.14 *(22.25)* | 51.76 *(30.87)* |
| Number of antenatal check-ups (mean, *SD*)* | 10.30 *(3.58)*  (*N* = 1509) | 10.86 *(3.25)*  (*N* = 464) | 10.05 *(3.69)*  (*N* = 1045) |
| Age at recruitment (years)  Mother age (mean*, SD)*  Mother less than 16 years *N* (%)  Father age categories *N* (%)  Under 16  Between 16 and 24  Between 25 and 34  Over 34  Missing | 17.83 *(1.24)*  113 (7.0)  45 (2.8)  1367 (84.5)  176 (10.9)  13 (0.8)  17 (1.1) | 17.91 *(1.22)*  32 (6.6)  14 (2.9)  408 (84.5)  56 (11.6)  2 (0.4)  3 (0.6) | 17.79 *(1.24)*  81 (7.1)  31 (2.7)  959 (84.5)  120 (10.6)  11 (1.0)  14 (1.2) |
| Ethnicity *N* (%)*  White background  Mixed background  Asian background  Black background  Other background | 1425 (88.1)  89 (5.5)  27 (1.7)  71 (4.4)  6 (0.4) | 436 (90.3)  29 (6.0)  8 (1.7)  10 (2.1)  0 (0.0) | 989 (87.1)  60 (5.3)  19 (1.7)  61 (5.4)  6 (1.5) |
| Language in the home *N* (%)*  English only  English and other language(s)  Other language(s) only | 1543 (95.4)  72 (4.4)  3 (0.2) | 469 (97.1)  12 (2.5)  2 (0.4) | 1074 (94.6)  60 (5.3)  1 (0.1) |
| NEET status^ *N* (%) |  |  |  |
| Yes  No  Participant age > 16 at baseline interview  Missing | 663 (41.0)  717 (44.3)  234 (14.5)  4 (0.2) | 190 (39.3)  231 (47.8)  62 (12.8)  0 (0.0) | 473 (41.7)  486 (42.8)  172 (15.2)  4 (0.4) |
| Index of Multiple Deprivation Score (IMD score) (mean*, SD)ᶧ* | 39.15 *(18.20)* | 38.73 *(18.01)* | 39.33 *(18.28)* |
| Relationship status with baby’s father *N* (%)  Married  Separated  Divorced  Closely involved/boyfriend  Just friends | 20 (1.2)  165 (10.2)  0 (0.0)  1222 (75.5)  211 (13.0) | 6 (1.2)  41 (8.5)  0 (0.0)  372 (77.0)  64 (13.3) | 14 (1.2)  124 (10.9)  0 (0.0)  850 (74.9)  147 (13.0) |

*Note*. *Significant differences detected between BABBLE sample and non-BABBLE sample (all *p*s < .05). ^Definition of NEET status: Not in education, employment or training (applicable only to those whose age at the end of previous academic year at time of baseline interview was > 16). *ᶧ*Higher IMD score indicates more deprivation.

Supplementary Table S2. *Bivariate analyses of family environment, maternal and child predictors of maternal MLU*

|  | Parameter estimate | 95% CI | p-value |
| --- | --- | --- | --- |
| Maternal age at recruitment (years) | 0.026 | -0.016 to 0.068 | 0.221 |
| Number of people live with at 24 months | 0.027 | -0.012 to 0.066 | 0.178 |
| Number of cigarettes 34-36 weeks gestation | -0.005 | -0.015 to 0.005 | 0.311 |
| NEET status at 24 months  No  Yes | Reference  -0.199 | -0.304 to -0.093 | 0.0001 |
| Relationship with child’s father at 24 monthsᵻ  Not in any relationship  Married  Separated  Closely involved/boyfriend  Just friends | Reference  -0.022  0.000  0.038  -0.008 | -0.324 to 0.281  -0.243 to 0.242  -0.085 to 0.161  -0.159 to 0.143 | 0.889  0.998  0.544  0.915 |
| Deprivation score at baseline | -0.002 | -0.005 to 0.001 | 0.125 |
| Psychological distress at 24 months | -0.003 | -0.010 to 0.004 | 0.452 |
| PND at 6 months | 0.007 | -0.005 to 0.020 | 0.227 |
| Social support at 24 months | 0.003 | 0.000 to 0.006 | 0.063 |
| Substance abuse at 24 months | 0.056 | -0.008 to 0.120 | 0.085 |
| Child MLU | 0.240 | 0.148 to 0.331 | 0.0001 |
| Sex of baby  Male  Female | Reference  0.109 | 0.008 to 0.211 | 0.035 |
| Age of child at 24 months | -0.005 | -0.042 to 0.032 | 0.793 |
| Birth weight (g) | 6.528E-5 | -3.052E-5 to 0.00 | 0.182 |
| Number of days breastfeeding | 0.002 | 0.001 to 0.004 | 0.001 |
| Number of weeks gestation at birth | 0.019 | -0.008 to 0.046 | 0.173 |

*Note.* Linear regressions. ^ᵻ^Only one participant in sample was divorced, so merged with ‘separated’ in the analysis.

NEET = Not in education, employment or training; PND = postnatal depression; MLU = mean length of utterance

Supplementary Table S3

*Bivariate analyses of family environment, maternal, and child predictors of children’s MLU*

|  | OR | 95% CI | p-value |
| --- | --- | --- | --- |
| Maternal age at recruitment | 1.13 | 0.97 to 1.31 | 0.111 |
| Number of people live with at 24 months | 0.93 | 0.81 to 1.07 | 0.332 |
| NEET status at 24 months  No  Yes | Reference  0.60 | 0.40 to 0.90 | 0.014 |
| Relationship with child’s father at 24 monthsᵻ  Not in any relationship  Married  Separated  Closely involved/boyfriend  Just friends | Reference  1.60  0.66  0.97  1.32 | 0.49 to 5.27  0.29 to 1.50  0.62 to 1.50  0.75 to 2.30 | 0.432  0.326  0.903  0.329 |
| Deprivation score at baseline | 1.00 | 0.99 to 1.01 | 0.491 |
| Psychological distress at 24 months | 0.97 | 0.95 to 1.00 | 0.081 |
| PND at 6 months | 1.01 | 0.97 to 1.06 | 0.461 |
| Social support at 24 months | 1.00 | 0.99 to 1.01 | 0.244 |
| Mother MLU | 1.86 | 1.32 to 2.63 | 0.000 |
| Sex of baby  Male  Female | Reference  2.53 | 1.73 to 3.70 | 0.000 |
| Age of baby at 24 month assessment | 1.10 | 0.96 to 1.27 | 0.149 |
| Substance abuse at 24 months | 0.87 | 0.70 to 1.09 | 0.222 |
| Birth weight | 1.00 | 1.00 to 1.01 | 0.086 |
| Number of days breastfeeding | 1.00 | 0.99 to 1.00 | 0.246 |
| Number of weeks gestation | 1.13 | 1.03 to 1.24 | 0.008 |
| Number of cigarettes 34-36 weeks | 0.99 | 0.95 to 1.02 | 0.675 |

*Note.* Ordinal regressions. ^ᵻ^Only one participant in sample was divorced, so merged with ‘separated’ in the analysis. PND = postnatal depression, MLU = mean length of utterance in morphemes.

**References**

AUTHORS. (2015).
